# Pheochromocytomas with Aggressive Histopathological Features Have Reduced Apelin and Increased VEGF Expression: Diagnostic and Prognostic Implications

**DOI:** 10.3390/pathophysiology33040072

**Published:** 2026-09-24

**Authors:** Hatice Çalışkan Burgucu, Tuğba Günler, Ethem Ömeroğlu, Yaşar Ünlü, Oğuzhan Aksu

**Affiliations:** 1 Division of Endocrinology and Metabolism, Department of Internal Medicine, Konya City Hospital, University of Health Sciences, Konya 42020, Turkey; droaksu@yahoo.com; 2 Department of Pathology, Konya City Hospital, Konya 42020, Turkey; tubagurcan85@hotmail.com; 3 Department of Pathology, Konya City Hospital, University of Health Sciences, Konya 42020, Turkey; patologethem@gmail.com (E.Ö.); yasarunlu66@gmail.com (Y.Ü.)

**Keywords:** pheochromocytoma, apelin, vascular endothelial growth factor, immunohistochemistry, PASS, GAPP

## Abstract

**Background:** Pheochromocytoma is a highly vascular neuroendocrine tumor with variable biological behavior. Reliable biomarkers reflecting tumor aggressiveness remain limited. This study evaluated apelin and vascular endothelial growth factor (VEGF) expression in pheochromocytoma and their associations with histopathological prognostic parameters. **Methods:** This retrospective single-center study included 20 pheochromocytoma patients and 18 normal adrenal medulla controls. Apelin and VEGF expression were evaluated immunohistochemically using the immunoreactivity score (IRS). Associations between IRS values and Pheochromocytoma of the Adrenal Gland Scaled Score (PASS), Grading System for Adrenal Pheochromocytoma and Paraganglioma (GAPP) score, Ki-67 index, tumor size, clinicopathological characteristics, and individual PASS components were analyzed. **Results:** Apelin IRS was significantly lower in pheochromocytoma than in normal adrenal medulla (median 0 vs. 4, *p* = 0.001), whereas VEGF IRS was significantly higher (median 6 vs. 2, *p* < 0.001). Tumors with PASS ≥ 4 had significantly lower apelin IRS than those with PASS < 4 (*p* = 0.001). Apelin IRS was inversely correlated with PASS (r = −0.874, *p* < 0.001), GAPP score (r = −0.618, *p* = 0.004), and Ki-67 (r = −0.560, *p* = 0.010). Lower apelin expression was also associated with diffuse growth pattern, central/confluent necrosis, and tumor cell spindling. VEGF IRS showed no significant association with PASS, GAPP score, Ki-67, tumor size, or individual PASS components. **Conclusions:** Reduced apelin immunoreactivity was associated with unfavorable histopathological features in pheochromocytoma, whereas increased VEGF immunoreactivity was not associated with the evaluated histopathological risk parameters. These exploratory findings suggest that apelin may be a candidate tissue marker; however, its clinical and prognostic relevance requires further investigation in larger multicenter studies.

## 1. Introduction

Pheochromocytomas and paragangliomas (PPGLs) are rare neuroendocrine neoplasms arising from chromaffin cells of the adrenal medulla and extra-adrenal paraganglia. According to the 2022 World Health Organization (WHO) Classification of Endocrine and Neuroendocrine Tumors, pheochromocytomas are regarded as intra-adrenal paragangliomas, and together with extra-adrenal paragangliomas are classified under the umbrella term PPGLs. Furthermore, the WHO no longer recommends the traditional benign–malignant distinction for PPGLs, as all tumors are considered to possess variable metastatic potential and no single histopathological feature can reliably predict metastatic behavior [1].

PPGLs are uncommon tumors with an annual incidence estimated at approximately 2–8 cases per million population. A recent systematic review evaluating epidemiological studies published over a 70-year period demonstrated that the incidence of PPGL has increased over time, reaching approximately 0.6 cases per 100,000 person-years in studies conducted after 2010, likely due to the widespread use of modern imaging modalities and increased incidental detection [2]. PPGLs occur most frequently between the third and fifth decades of life and affect men and women with similar frequency [3].

Given the heterogeneous clinical behavior of PPGLs, several histopathological scoring systems have been developed to estimate metastatic risk and biological aggressiveness. The Pheochromocytoma of the Adrenal Gland Scaled Score (PASS) is based on histopathological features and is primarily applied to pheochromocytomas, with scores ≥ 4 generally considered indicative of increased aggressive potential. The Grading System for Adrenal Pheochromocytoma and Paraganglioma (GAPP) incorporates histological characteristics, Ki-67 proliferation index, and catecholamine phenotype to stratify tumors according to metastatic risk [4]. However, despite their widespread use in clinical practice, the 2022 WHO classification does not endorse any single scoring system as a definitive predictor of metastatic behavior [1,5]. Despite advances in the pathological and molecular characterization of PPGLs, reliable biomarkers that reflect tumor aggressiveness and histopathological risk remain limited. The identification of novel biological markers may improve risk stratification and provide further insights into the mechanisms underlying tumor progression.

Apelin is an endogenous peptide ligand of the G protein-coupled APJ receptor that was first identified by Tatemoto et al. in 1998 [6]. The apelin/APJ signaling pathway is widely expressed in multiple tissues and has been implicated in numerous physiological processes, including cardiovascular regulation, angiogenesis, fluid homeostasis, energy metabolism, and neuroendocrine function [7]. Increasing evidence indicates that the apelin/APJ axis also plays an important role in tumor biology. Through its effects on angiogenesis, cell proliferation, migration, invasion, and tumor microenvironment remodeling, apelin has been associated with tumor progression and adverse prognostic features in several malignancies [7,8]. Recent studies further suggest that apelin signaling contributes to tumor vascularization and neovascularization, highlighting its potential value as a diagnostic, prognostic, and therapeutic target in cancer [8,9]. Emerging evidence suggests that apelin and VEGF may act synergistically during tumor angiogenesis, with apelin enhancing endothelial cell proliferation and promoting VEGF-mediated neovascularization [10].

Vascular endothelial growth factor (VEGF) is a potent endothelial cell-specific mitogen and one of the principal regulators of physiological and pathological angiogenesis. Since its identification, VEGF has been recognized as a critical mediator of tumor neovascularization, promoting endothelial cell proliferation, migration, survival, and vascular permeability through activation of its tyrosine kinase receptors, particularly vascular endothelial growth factor receptors 1 and 2 (VEGFR-1 and VEGFR-2) [11]. The VEGF signaling pathway plays a fundamental role in tumor growth by facilitating the formation of new blood vessels that supply oxygen and nutrients to rapidly proliferating neoplastic cells. In addition to supporting tumor expansion, VEGF contributes to local invasion and metastatic dissemination by increasing vascular permeability and remodeling the tumor microenvironment [12].

Overexpression of VEGF has been demonstrated in a wide variety of solid malignancies, including ovarian, gastric, breast, renal, colorectal, and lung cancers, where elevated VEGF levels have frequently been associated with increased tumor aggressiveness, advanced disease stage, higher metastatic potential, and poorer clinical outcomes [13,14,15,16]. Because angiogenesis is essential for the progression of highly vascular tumors, VEGF expression has attracted considerable interest as both a prognostic biomarker and a potential therapeutic target. Indeed, anti-angiogenic therapies targeting the VEGF pathway have become an established treatment strategy in several malignancies [17].

Pheochromocytomas are highly vascular neuroendocrine tumors characterized by a rich capillary network and marked expression of angiogenesis-related factors. The highly vascular nature of PPGLs is largely driven by pseudohypoxia-related molecular pathways involving hypoxia-inducible factor (HIF) stabilization and downstream activation of angiogenic mediators such as VEGF. Previous studies have suggested that dysregulation of angiogenic pathways may contribute to the biological behavior of pheochromocytomas and paragangliomas; however, the prognostic significance of VEGF expression in these tumors remains incompletely understood. Therefore, evaluation of VEGF expression may provide valuable insights into the mechanisms underlying tumor progression and may help identify histopathological features associated with aggressive clinical behavior [18,19].

Considering the established roles of apelin and VEGF in tumor angiogenesis and progression, we aimed to evaluate the expression of apelin and VEGF in pheochromocytoma tissues and investigate their associations with clinicopathological characteristics, PASS, GAPP score, Ki-67 proliferation index, and biochemical features of the tumors.

## 2. Materials and Methods

### 2.1. Study Population

This retrospective, single-center study included patients who underwent adrenalectomy for pheochromocytoma at Konya City Hospital between 24 August 2020 and 1 March 2026. The diagnosis of pheochromocytoma was established based on compatible clinical manifestations, elevated plasma free metanephrine and/or normetanephrine concentrations or 24 h urinary fractionated metanephrine levels, cross-sectional imaging (computed tomography [CT] and/or magnetic resonance imaging [MRI]), and histopathological confirmation following adrenalectomy. A total of 20 patients with histopathologically confirmed pheochromocytoma were included in the study. The control group consisted of 18 histologically normal adrenal medulla samples, of which 15 were obtained from nephrectomy specimens of patients undergoing surgery for non-adrenal diseases and 3 from adrenal medulla adjacent to benign adrenal lesions. The control adrenal medulla was confirmed to be histologically normal on pathological examination. None of the control patients had a known adrenal or endocrine tumor-related condition that could potentially influence apelin or VEGF immunoreactivity. Patients with recurrent PPGL, adrenal cortical tumors, metastatic adrenal tumors, insufficient tissue for immunohistochemical evaluation, or incomplete clinicopathological data were excluded. Demographic, biochemical, radiological, operative, and clinicopathological data were retrospectively obtained from the electronic medical records. Information regarding germline genetic testing, including RET mutation analysis and multiple endocrine neoplasia type 2 (MEN2)-associated genetic evaluation, was not consistently available in the retrospective medical records; therefore, MEN2 status could not be systematically assessed in the present study.

The study was approved by the relevant institutional ethics committee, and all patient data were anonymized before analysis owing to the retrospective design.

### 2.2. Histopathological Evaluation

Archived formalin-fixed, paraffin-embedded pheochromocytoma specimens were retrieved from the Department of Pathology. All hematoxylin–eosin stained slides were independently reviewed by experienced pathologists.

Histopathological risk stratification was performed using both the Pheochromocytoma of the Adrenal Gland Scaled Score (PASS) and the Grading System for Adrenal Pheochromocytoma and Paraganglioma (GAPP). PASS was calculated according to the original scoring system, which evaluates vascular invasion, capsular invasion, periadrenal adipose tissue invasion, diffuse or large nest growth pattern, focal or confluent necrosis, high cellularity, tumor cell spindling, cellular monotony, increased mitotic activity, atypical mitotic figures, nuclear pleomorphism, and hyperchromasia, yielding a maximum score of 20. Tumors with a PASS ≥ 4 were considered to have increased histopathological risk [20]. The GAPP score was determined according to the original grading system based on histological pattern, cellularity, comedo-type necrosis, capsular and/or vascular invasion, Ki-67 labeling index, and catecholamine phenotype [21]. Associations of apelin and VEGF immunohistochemical expression with total PASS, total GAPP score, individual PASS parameters, Ki-67 proliferation index, and catecholamine phenotype were evaluated. Comparative analyses were also performed according to PASS categories (≥4 vs. <4).

### 2.3. Immunohistochemical Analysis

Formalin-fixed, paraffin-embedded tissue blocks from pheochromocytoma and control specimens were used for immunohistochemical evaluation. Serial sections of 4–5 µm thickness were obtained from each paraffin block and mounted on positively charged slides. Immunohistochemical staining was performed using a closed-system automated staining platform (DAKO, Glostrup, Denmark) according to the manufacturer’s instructions.

One slide was incubated with a primary antibody against VEGF (RB-9031-PO, Thermo Fisher Scientific, Waltham, MA, USA), and the other with a primary antibody against apelin (Apelin Polyclonal Antibody, orb1411607, Biorbyt Ltd., Cambridge, UK). For VEGF immunohistochemistry, heat-induced epitope retrieval (HIER) was performed in 1 mM ethylenediaminetetraacetic acid (EDTA) buffer (pH 8.0) at boiling temperature for 10–20 min, followed by cooling at room temperature for 20 min. The VEGF primary antibody was applied at a dilution of 1:100 and incubated for 30 min at room temperature. For apelin immunohistochemistry, HIER was performed in citrate buffer (pH 6.0) at boiling temperature for 15 min, followed by cooling at room temperature. The apelin primary antibody was applied at a dilution of 1:200 and incubated overnight at 4 °C. Immunohistochemical staining was performed using the DAKO Autostainer Link 48 automated staining platform (Dako/Agilent Technologies, Glostrup, Denmark), with the EnVision FLEX detection/visualization system. 3,3′-Diaminobenzidine (DAB) was used as the chromogen, and hematoxylin was used for counterstaining. Placental tissue was used as a positive control for both apelin and VEGF, with strong cytoplasmic immunoreactivity in villous trophoblasts and intravillous vascular endothelial cells considered indicative of appropriate staining. Negative controls were processed using the same immunohistochemical procedure with omission of the primary antibody, and no specific immunoreactivity was observed. Brown cytoplasmic immunoreactivity was considered indicative of positive expression for both antibodies.

Each slide was initially scanned in its entirety at low magnification. A fixed number of high-power fields (HPFs) was not used. Representative areas of viable tumor tissue without necrosis, hemorrhage, or technical artifacts were evaluated at ×200 and ×400 magnification to determine the percentage of positively stained cells and staining intensity.

### 2.4. Immunohistochemical Scoring

For each specimen, immunoreactivity was evaluated semi-quantitatively using the immunoreactivity score (IRS) method by separately assessing the proportion of positively stained cells and staining intensity. The proportion of positively stained cells was graded as follows: 0 (no positive cells), 1 (<25% positive cells), 2 (25–50% positive cells), 3 (51–75% positive cells), and 4 (>75% positive cells). Staining intensity was independently scored as 0 (negative), 1 (weak), 2 (moderate), or 3 (strong) [22].

The IRS was calculated for each specimen by multiplying the proportion score by the staining intensity score, yielding a total score ranging from 0 to 12, with higher IRS values indicating greater immunoreactivity. According to the IRS values, immunostaining was categorized as negative (0–1), weak (2–3), moderate (4–8), or strong (9–12). For statistical analyses, IRS values were analyzed as continuous variables rather than as categorical staining groups [23].

### 2.5. Statistical Analysis

Statistical analyses were performed using IBM SPSS Statistics, version 26.0 (IBM Corp., Armonk, NY, USA). The normal distribution of continuous variables was assessed using the Shapiro–Wilk test. As the majority of continuous variables did not follow a normal distribution, non-parametric tests were used throughout the analysis; continuous variables were expressed as median interquartile range (IQR) and categorical variables as *n* (%). Differences between the pheochromocytoma and control groups were evaluated using the Mann–Whitney U test for continuous variables and Fisher’s exact test for categorical variables. Within the pheochromocytoma group, associations between apelin/VEGF IRS and continuous histopathological parameters (tumor size, Ki-67 index, total GAPP score, total PASS, and individual PASS components) were assessed using Spearman’s correlation analysis. Comparisons of apelin and VEGF IRS according to two-category histopathological and clinical parameters, including PASS category (<4 vs. ≥4), were performed using the Mann–Whitney U test, and comparisons across variables with three categories (tumor localization, catecholamine phenotype) were performed using the Kruskal–Wallis H test. A *p* value < 0.05 was considered statistically significant.

## 3. Results

A total of 20 patients with pheochromocytoma and 18 control subjects with normal adrenal medulla tissue were included in the study. Demographic characteristics were similar between the pheochromocytoma and control groups; median age was 51 (IQR, 37–58) years in the pheochromocytoma group and 50.5 (IQR, 38–60) years in the control group (*p* = 1.000), and the proportion of female subjects did not differ significantly between the groups (55.0% vs. 66.7%, *p* = 0.463) (Table 1).

Within the pheochromocytoma group, tumors were located in the right adrenal gland in 50.0% of cases, in the left adrenal gland in 40.0%, and bilaterally in 10.0%. According to catecholamine phenotype, 75.0% of tumors were noradrenergic-predominant, 15.0% were adrenergic-predominant, and 10.0% were dopaminergic-predominant. The median tumor size was 5.2 cm (IQR, 4.0–6.1), the median Ki-67 proliferation index was 3% (IQR, 1.8–5), the median total GAPP score was 5 (IQR, 4–6), and the median total PASS was 6 (IQR, 2.3–8.8) (Table 1).

Apelin IRS was significantly lower in the pheochromocytoma group than in the control group [0 (IQR, 0–2) vs. 4 (IQR, 1–6); *p* = 0.001]. In contrast, VEGF IRS was significantly higher in the pheochromocytoma group than in the control group [6 (IQR, 4–10.5) vs. 2 (IQR, 2–2); *p* < 0.001] (Table 1). Representative immunohistochemical staining patterns of apelin and VEGF in normal adrenal medulla and pheochromocytoma tissues are shown in Figure 1.

When patients were dichotomized according to the established PASS threshold, apelin IRS was significantly lower in tumors with a PASS ≥ 4 than in those with a PASS < 4 [0 (IQR, 0–0) vs. 2 (IQR, 2–3); *p* = 0.001]. VEGF IRS did not differ significantly between the two groups [6 (IQR, 4–10.5) vs. 5 (IQR, 4–9); *p* = 0.792] (Table 2).

Median apelin IRS was 1.5 (IQR, 0–2) in right-sided tumors, 0 (IQR, 0–0.5) in left-sided tumors, and 0 (IQR, 0–0) in bilateral tumors, without a significant difference (*p* = 0.253). Likewise, VEGF IRS did not differ significantly according to tumor localization (median [IQR]: 4 [4–10.5], 6 [4,5,6,7,8,9], and 9 [7.5–10.5], respectively; *p* = 0.489). Neither apelin nor VEGF IRS differed according to catecholamine phenotype (apelin: *p* = 0.277; VEGF: *p* = 0.803). Similarly, hypertension status was not associated with either apelin IRS (*p* = 0.082) or VEGF IRS (*p* = 1.000) (Table 3). Spearman correlation analysis showed that apelin IRS was significantly and negatively correlated with total PASS (r = −0.874, *p* < 0.001), total GAPP score (r = −0.618, *p* = 0.004), and Ki-67 index (r = −0.560, *p* = 0.010). No significant correlations were observed between apelin IRS and tumor size (r = −0.309, *p* = 0.184) or VEGF IRS (r = −0.110, *p* = 0.645). VEGF IRS was not significantly correlated with tumor size (r = −0.028, *p* = 0.905), Ki-67 index (r = 0.194, *p* = 0.414), total GAPP score (r = −0.034, *p* = 0.886), or total PASS (r = 0.002, *p* = 0.993) (Table 4).

To allow direct comparison with the correlation-based approach used in previous literature, associations between apelin/VEGF IRS and the individual components of the PASS were additionally examined using Spearman’s correlation analysis. Apelin IRS was significantly negatively correlated with the presence of a diffuse growth pattern (*r* = −0.874, *p* < 0.001), central/confluent tumor necrosis (*r* = −0.457, *p* = 0.043), and tumor cell spindling (*r* = −0.457, *p* = 0.043). No significant correlation was found between apelin IRS and the remaining PASS components, including high cellularity, increased mitotic activity, atypical mitotic figures, periadrenal adipose tissue invasion, vascular invasion, capsular invasion, nuclear pleomorphism, and nuclear hyperchromasia (all *p* > 0.05). VEGF IRS showed no significant correlation with any individual PASS component (all *p* > 0.05) (Table 5). Cellular monotony was not evaluated because this parameter was uniformly absent across all tumors.

## 4. Discussion

In the present study, apelin immunoreactivity was significantly lower, whereas VEGF immunoreactivity was significantly higher, in pheochromocytoma tissues than in normal adrenal medulla. Lower apelin IRS was inversely associated with PASS and GAPP scores and the Ki-67 index. Tumors with a PASS ≥ 4 also exhibited lower apelin IRS than tumors with a PASS < 4. Nominal inverse associations were additionally observed between apelin IRS and specific PASS components, including diffuse growth pattern, central/confluent necrosis, and tumor cell spindling; however, these component-level findings should be interpreted cautiously because no formal adjustment for multiple comparisons was applied. In contrast, VEGF IRS was not associated with the evaluated histopathological risk parameters. Collectively, these findings indicate that reduced apelin immunoreactivity is associated with unfavorable histopathological features in pheochromocytoma, whereas increased VEGF immunoreactivity was not associated with histopathological features of aggressive behavior in this cohort.

Angiogenesis is a fundamental hallmark of tumor development, providing the vascular network required to sustain tumor growth, local invasion, and metastatic dissemination by ensuring an adequate supply of oxygen and nutrients [24]. This process is largely driven by hypoxia within the tumor microenvironment, which induces the expression of multiple pro-angiogenic mediators and their receptors [25]. Among these, VEGF is considered the principal regulator of tumor angiogenesis, promoting endothelial cell proliferation, migration, survival, and vascular permeability through activation of VEGF receptors [26,27]. In parallel, accumulating evidence indicates that the apelin/APJ signaling pathway also contributes to tumor vascularization by stimulating endothelial cell proliferation, vascular remodeling, and neovascularization. Experimental studies further suggest that apelin may enhance VEGF-mediated angiogenic responses, supporting the concept that these two signaling pathways act in a complementary rather than independent manner during tumor angiogenesis [8,9,10]. However, these mechanistic interactions have primarily been demonstrated in experimental models and cannot be inferred directly from tissue immunoreactivity alone.

Pheochromocytomas are highly vascular neuroendocrine tumors with a dense and complex vascular network that can be readily identified by contrast-enhanced CT and MRI, particularly in larger lesions [28]. This pronounced vascularity is not only a characteristic imaging feature but also has important clinical implications, as patients undergoing adrenalectomy for pheochromocytoma have an increased risk of intraoperative blood transfusion and postoperative cardiopulmonary complications compared with patients undergoing adrenalectomy for other hormonally active adrenal tumors [29].

Evidence regarding angiogenesis-related biomarkers in pheochromocytoma remains limited [30]. Earlier studies using endothelial markers such as factor VIII and CD34 demonstrated increased microvessel density in pheochromocytoma; however, these markers showed limited value in predicting metastatic behavior or malignant potential [31,32]. Among angiogenic biomarkers, VEGF is the most well-characterized factor and plays a central role in tumor angiogenesis [30]. Previous studies have consistently demonstrated higher VEGF expression in pheochromocytoma compared with normal adrenal medulla, particularly in tumors with metastatic or aggressive histopathological features [33,34]. More recently, Abe et al. evaluated the expression of VEGF-A, VEGF-B, and VEGF-C in non-hereditary, non-metastatic pheochromocytomas and reported frequent expression of all three VEGF isoforms, supporting the concept that activation of the VEGF pathway is a characteristic feature even in clinically localized tumors. These findings further emphasize that VEGF overexpression is an intrinsic biological characteristic of pheochromocytoma rather than a feature restricted to metastatic disease [35]. Similarly, Kocabaş et al. also reported significantly higher VEGF immunoreactivity in pheochromocytoma tissues and observed positive associations between VEGF expression, PASS, and Ki-67 index, suggesting that VEGF may reflect adverse histopathological characteristics [36]. Taken together, previous studies consistently support increased VEGF expression in pheochromocytoma, although its relationship with histopathological aggressiveness remains controversial [36,37].

Consistent with these findings, VEGF expression was significantly higher in pheochromocytoma than in normal adrenal medulla in our cohort. However, unlike previous reports, VEGF IRS was not significantly associated with PASS, GAPP score, Ki-67 index, or tumor size. Moreover, no significant correlation was observed between apelin IRS and VEGF IRS in our cohort. Therefore, although interactions between the apelin/APJ and VEGF pathways have been proposed in experimental studies [9,38], our immunohistochemical findings do not demonstrate coordinated expression of these markers in pheochromocytoma tissue. These discrepancies may reflect differences in cohort characteristics, sample size, immunohistochemical scoring methods, and the relatively limited number of tumors with aggressive pathological features included in the respective studies. Nevertheless, our findings indicate that VEGF immunoreactivity was higher in pheochromocytoma than in normal adrenal medulla but was not associated with the evaluated histopathological features of aggressive behavior. Because direct measures of vascularity and angiogenesis were not performed, increased VEGF immunoreactivity should not be interpreted as direct evidence of increased angiogenic activity.

Apelin, the endogenous ligand of the G protein-coupled APJ receptor, is increasingly recognized as a key regulator of physiological and pathological angiogenesis [8]. Activation of the apelin/APJ signaling pathway promotes endothelial cell proliferation, migration, and vascular sprouting through both VEGF-dependent and VEGF-independent mechanisms, highlighting its pivotal role in vascular remodeling [8,39,40]. In cancer, the apelin/APJ axis has been implicated in tumor angiogenesis, cell proliferation, invasion, and modulation of the tumor microenvironment [41]. Although most solid tumors demonstrate increased apelin expression associated with tumor progression, recent evidence suggests that the biological role of apelin is highly tumor-specific and depends on the tumor microenvironment [8]. Increased apelin expression has been reported in a variety of solid malignancies, including lung, hepatocellular, colorectal, breast, ovarian, and prostate cancers, where it has frequently been associated with enhanced tumor progression and poor clinical outcomes [42]. However, evidence regarding apelin expression in pheochromocytoma remains scarce [43]. In the only immunohistochemical study evaluating apelin in pheochromocytoma and paraganglioma (PPGL), Wang et al. demonstrated significantly lower apelin expression in metastatic compared with non-metastatic tumors, while negative apelin expression was associated with significantly shorter metastasis-free survival, suggesting that loss of apelin expression may be associated with a more aggressive biological phenotype [43]. Consistent with these observations, lower apelin expression in our cohort was associated with higher PASS and GAPP scores and increased Ki-67 index. Our findings further extend these observations by demonstrating that reduced apelin expression was associated not only with higher PASS and GAPP scores and increased Ki-67 index, but also with adverse histopathological features including diffuse growth pattern, central/confluent necrosis, and tumor cell spindling, supporting its potential role as an immunohistochemical marker associated with unfavorable histopathological features in pheochromocytoma.

Evidence from studies including both pheochromocytomas and paragangliomas provides some insight into the potential relevance of these markers across the PPGL spectrum. Wang et al. evaluated apelin expression in both pheochromocytomas and paragangliomas and reported lower apelin expression in metastatic than in non-metastatic PPGLs, suggesting a possible association between reduced apelin expression and unfavorable tumor behavior [43]. Günler et al. evaluated VEGF expression in pheochromocytomas and intra-abdominal paragangliomas and found no significant association between VEGF expression and GAPP score, PASS, or histopathological differentiation, in line with our findings regarding VEGF [37]. However, paragangliomas were not included in the present study; therefore, whether the associations observed for apelin and VEGF also apply to paragangliomas remains uncertain. Further studies specifically including paragangliomas are needed to clarify this issue.

The clinical relevance of tumor laterality in pheochromocytoma remains uncertain. Previous studies have suggested that right-sided pheochromocytomas may differ from left-sided tumors in certain clinical characteristics and recurrence risk [44]. However, a more recent study of hereditary pheochromocytoma found that tumor laterality was not associated with recurrence risk [45]. In our cohort, neither apelin nor VEGF immunoreactivity differed significantly according to tumor localization, suggesting no apparent association between laterality and the expression of these markers.

Although PASS and GAPP remain the most widely used histopathological scoring systems for risk assessment in pheochromocytoma, their prognostic performance is imperfect and considerable interobserver variability has been reported [5,46]. Consequently, current research increasingly focuses on identifying complementary molecular and immunohistochemical biomarkers that may improve biological risk stratification [47]. In this context, the strong inverse associations observed between apelin expression and both PASS and GAPP scores suggest that apelin may provide additional biological information beyond conventional histopathological assessment rather than replacing existing scoring systems. However, the development and validation of a composite scoring system integrating apelin immunoreactivity with established histopathological parameters would require larger, independent cohorts with longitudinal outcome data and is beyond the scope of the present study.

The biological mechanisms underlying the inverse association between apelin expression and histopathological aggressiveness in pheochromocytoma remain uncertain. Experimental studies have shown that the apelin/APJ signaling pathway regulates multiple intracellular cascades, including PI3K/Akt, ERK1/2, AMPK, and nitric oxide signaling, which collectively influence endothelial function, angiogenesis, cell survival, and cellular metabolism [9,48]. Furthermore, interactions between apelin signaling and the HIF-1α/VEGF axis have been described under hypoxic conditions, suggesting that apelin participates in the adaptive response of tissues to oxygen deprivation [9]. However, the functional consequences of apelin activation appear to vary substantially according to tumor type, cellular origin, and the surrounding tumor microenvironment, indicating that apelin may exert either tumor-promoting or tumor-restraining effects depending on the biological context [41].

The observed reduction in apelin immunoreactivity in tumors with higher PASS and GAPP scores may reflect underlying biological differences associated with unfavorable histopathological characteristics. Alterations in neuroendocrine differentiation, hypoxia-associated signaling, cellular metabolism, or stromal interactions could potentially influence apelin/APJ expression, as suggested by previous experimental and tumor-related studies [41,42,43]; however, these mechanisms were not directly investigated in the present study. The inverse correlation between apelin IRS and the Ki-67 index suggests an association between reduced apelin immunoreactivity and increased proliferative activity. Nevertheless, owing to the cross-sectional tissue-based design and the absence of complementary molecular and longitudinal clinical data, reduced apelin expression cannot be interpreted as evidence of tumor progression or as a causal contributor to aggressive behavior.

To our knowledge, this is among the few studies evaluating the relationship between apelin expression and established histopathological prognostic scoring systems in pheochromocytoma. These inverse associations suggest that apelin immunoreactivity may provide complementary information regarding unfavorable histopathological features; however, its incremental value beyond established scoring systems remains to be determined.

### Limitations

The present study has several limitations. First, its retrospective, single-center design and relatively small sample size limited the statistical power of the analyses, particularly the subgroup analyses, and may restrict the generalizability of the findings. Given the exploratory nature of the study, no formal prespecified primary endpoint was defined, and multiple comparisons were performed without formal adjustment for multiplicity. Therefore, the statistically significant associations should be interpreted cautiously as nominal exploratory findings. Second, clinically relevant longitudinal outcomes, including metastatic progression, recurrence, disease-free survival, and disease-specific survival, were unavailable. Consequently, PASS, GAPP, and Ki-67 were evaluated as histopathological features associated with aggressive behavior rather than as direct measures of metastatic potential or clinical prognosis. Genetic data, including RET mutation analysis for MEN2 and other PPGL susceptibility genes such as SDHB, were not consistently available in the retrospective medical records, precluding systematic assessment of hereditary syndromes and their potential implications for patients and family members. Quantitative catecholamine levels were not available for all patients, precluding a reliable assessment of their correlations with histopathological parameters of aggressiveness. In addition, only two patients exhibited a dopaminergic-predominant biochemical phenotype, precluding meaningful subgroup analyses or conclusions regarding the association of this uncommon phenotype with apelin or VEGF immunoreactivity. Another limitation is that apelin and VEGF expression were assessed using semi-quantitative immunohistochemistry, which, despite blinded evaluation by three experienced pathologists and consensus scoring, remains an observer-dependent technique. Complementary quantitative analyses, including gene or protein expression assays, APJ/APLNR and VEGF receptor expression, circulating apelin measurements, and direct measures of angiogenesis such as microvessel density or endothelial markers including CD31 and CD34, were not performed. Moreover, the cross-sectional tissue-based design does not permit causal inference regarding the relationship between reduced apelin expression and histopathological features of aggressive behavior. Accordingly, the present findings should be considered exploratory and hypothesis-generating and interpreted with appropriate caution until they are confirmed in larger, multicenter studies incorporating longitudinal clinical outcomes, genetic characterization, and complementary quantitative molecular approaches.

## 5. Conclusions

In conclusion, pheochromocytoma tissues exhibited significantly lower apelin immunoreactivity and higher VEGF immunoreactivity than normal adrenal medulla. Lower apelin IRS was inversely associated with PASS and GAPP scores and the Ki-67 index, whereas VEGF IRS was not associated with the evaluated histopathological features of aggressive behavior. These exploratory findings suggest that reduced apelin immunoreactivity may be a candidate tissue marker associated with unfavorable histopathological characteristics in pheochromocytoma. However, the present study does not establish its prognostic value or a causal relationship with tumor progression. Furthermore, because direct measures of vascularity and angiogenesis were not performed, higher VEGF immunoreactivity should not be interpreted as direct evidence of increased angiogenic activity. Larger multicenter studies incorporating longitudinal clinical outcomes and complementary quantitative molecular analyses are required to determine the clinical and biological relevance of these findings.

## Figures and Tables

**Figure 1:**
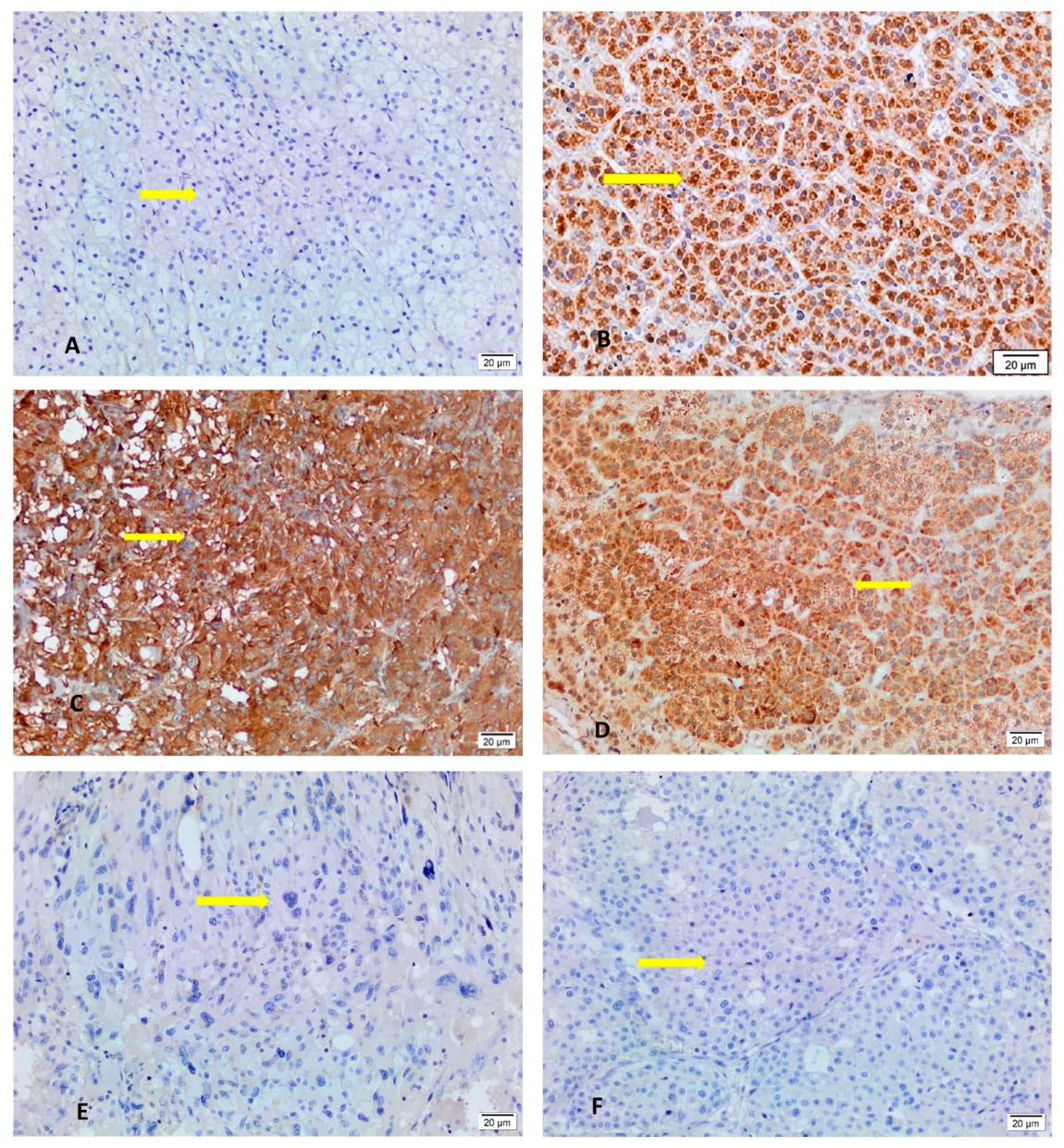
Representative immunohistochemical staining of vascular endothelial growth factor (VEGF) and apelin in normal adrenal medulla and pheochromocytoma tissues. (**A**) VEGF negative in normal adrenal medulla tissue (×200); (**B**). Apelin expression in normal adrenal medulla tissue (×200); (**C**) strong VEGF expression in pheochromocytoma (×200); (**D**) strong apelin expression in pheochromocytoma (×200); (**E**) negative VEGF expression in pheochromocytoma (×200); (**F**) negative apelin expression in pheochromocytoma (×200). Yellow arrows indicate representative cells showing the described immunoreactivity.

**Table 1:** Demographic, clinical, and histopathological characteristics.

Variable	Pheochromocytoma (*n* = 20)	Control (*n* = 18)	*p*
Age, years, median (IQR)	51 (37–58)	50.5 (38–60)	1.000
Sex, female, *n* (%)	11 (55.0)	12 (66.7)	0.463
Apelin IRS, median (IQR)	0 (0–2)	4 (1–6)	0.001
VEGF IRS, median (IQR)	6 (4–10.5)	2 (2–2)	<0.001
**Tumor characteristics (pheochromocytoma group, *n* = 20)**			
Tumor size, cm, median (IQR)	5.2 (4.0–6.1)		
Tumor localization, *n* (%)			
Right adrenal	10 (50.0)		
Left adrenal	8 (40.0)		
Bilateral	2 (10.0)		
Catecholamine phenotype, *n* (%)			
Noradrenergic-predominant	15 (75.0)		
Adrenergic-predominant	3 (15.0)		
Dopaminergic-predominant	2 (10.0)		
Ki-67 index, %, median (IQR)	3 (1.8–5)		
Total GAPP score, median (IQR)	5 (4–6)		
Total PASS, median (IQR)	6 (2.3–8.8)		
Hypertension, *n* (%)	8 (40.0)		

Statistical analysis: Continuous variables were compared using the Mann–Whitney U test, and categorical variables were compared using Fisher’s exact test. Data are presented as median (IQR) or *n* (%). Abbreviations: IQR, interquartile range; IRS, immunoreactivity score; VEGF, vascular endothelial growth factor; PASS, Pheochromocytoma of the Adrenal Gland Scaled Score; GAPP, Grading System for Adrenal Pheochromocytoma and Paraganglioma. Bold text indicates section headings within the table.

**Table 2:** Apelin and VEGF IRS according to PASS category in the pheochromocytoma group (*n* = 20).

Variable	PASS < 4 (*n* = 8)	PASS ≥ 4 (*n* = 12)	*p*
Apelin IRS, median (IQR)	2 (2–3)	0 (0–0)	0.001
VEGF IRS, median (IQR)	5 (4–9)	6 (4–10.5)	0.792

Data are presented as median (IQR). PASS: Pheochromocytoma of the Adrenal Gland Scaled Score. *p* values were calculated using the Mann–Whitney U test.

**Table 3:** Comparison of apelin and VEGF immunoreactivity scores according to clinicopathological characteristics in patients with pheochromocytoma (*n* = 20).

Variable	*n*	Apelin IRS, Median (IQR)	VEGF IRS, Median (IQR)	*p* Apelin	*p* VEGF
***Tumor localization***					
Right adrenal	10	1.5 (0–2)	4 (4–10.5)	0.253	0.489
Left adrenal	8	0 (0–0.5)	6 (4–9)		
Bilateral	2	0 (0–0)	9 (7.5–10.5)		
***Catecholamine phenotype***					
Noradrenergic-predominant	15	0 (0–2)	6 (4–9)	0.277	0.803
Adrenergic-predominant	3	2 (1–3)	4 (3–8)		
Dopaminergic-predominant	2	0 (0–0)	8 (6–10)		
***Hypertension***					
Absent	12	1.5 (0–2)	6 (4–9.8)	0.082	1.000
Present	8	0 (0–0)	5 (4–9.8)		

Statistical analysis: Comparisons among three groups were performed using the Kruskal–Wallis test, whereas comparisons between two groups were performed using the Mann–Whitney U test. Data are presented as median (IQR) or *n* (%). Abbreviations: IQR, interquartile range; IRS, immunoreactivity score; VEGF, vascular endothelial growth factor. Bold italic text indicates section headings within the table.

**Table 4:** Spearman correlation analysis between apelin/VEGF IRS and histopathological parameters in the pheochromocytoma group (*n* = 20).

	Apelin IRS		VEGF IRS	
Variable	*r*	*p*	*r*	*p*
VEGF IRS	−0.110	0.645	—	—
Tumor size	−0.309	0.184	−0.028	0.905
Ki-67 index	−0.560	0.010	0.194	0.414
Total GAPP score	−0.618	0.004	−0.034	0.886
Total PASS	−0.874	<0.001	0.002	0.993

Statistical analysis: Spearman’s correlation analysis. Abbreviations: IRS, immunoreactivity score; VEGF, vascular endothelial growth factor; PASS, Pheochromocytoma of the Adrenal Gland Scaled Score; GAPP, Grading System for Adrenal Pheochromocytoma and Paraganglioma; r, Spearman correlation coefficient.

**Table 5:** Spearman correlation analysis between apelin/VEGF IRS and individual PASS components in the pheochromocytoma group (*n* = 20).

	Apelin IRS		VEGF IRS	
PASS Component	*r*	*p*	*r*	*p*
Large nests or diffuse growth (>10% of tumor volume)	−0.874	<0.001	−0.072	0.764
Central or confluent tumor necrosis	−0.457	0.043	0.226	0.338
High cellularity	−0.262	0.264	0.191	0.421
Tumor cell spindling	−0.457	0.043	−0.021	0.932
Greater than 3 mitotic figures/10 high-power field	−0.395	0.084	0.044	0.852
Atypical mitotic figures	−0.264	0.261	0.059	0.804
Extension into periadrenal adipose tissue	−0.264	0.261	−0.341	0.141
Vascular invasion	−0.160	0.501	−0.103	0.667
Capsular invasion	−0.131	0.581	−0.236	0.316
Profound nuclear pleomorphism	−0.415	0.069	−0.308	0.187
Nuclear hyperchromasia	−0.238	0.311	−0.125	0.599

Statistical analysis: Spearman’s correlation analysis. Abbreviations: IRS, immunoreactivity score; VEGF, vascular endothelial growth factor; PASS, Pheochromocytoma of the Adrenal Gland Scaled Score; r, Spearman correlation coefficient. The cellular monotony component was excluded from the correlation analysis because it was uniformly absent (score = 0) in all cases.

## Data Availability

The datasets generated and/or analyzed during the current study are not publicly available due to patient confidentiality and institutional policies but are available from the corresponding author upon reasonable request.

## References

[1] Mete O., Asa S.L., Gill A.J., Kimura N., de Krijger R.R., Tischler A. (2022). Overview of the 2022 WHO Classification of Paragangliomas and Pheochromocytomas. Endocr. Pathol..

[2] Al Subhi A.R., Boyle V., Elston M.S. (2022). Systematic Review: Incidence of Pheochromocytoma and Paraganglioma Over 70 Years. J. Endocr. Soc..

[3] Melli B., Cusenza V.Y., Martinelli S., Castiglione F., Fornaciari L., Palicelli A., Braglia L., Farnetti E., Negro A., Rosato S. (2025). Clinical exome next-generation sequencing panel for hereditary pheochromocytoma and paraganglioma diagnosis. Exp. Ther. Med..

[4] Bima C., Bioletto F., Lopez C., Bollati M., Arata S., Procopio M., Gesmundo I., Ghigo E., Maccario M., Parasiliti-Caprino M. (2022). Clinical and Pathological Tools for Predicting Recurrence and/or Metastasis in Patients with Pheochromocytoma and Paraganglioma. Biomedicines.

[5] Li Q., Lan Z., Jiang Y., Wang R., Li Z., Jiang X. (2024). Validation and Evaluation of 5 Scoring Systems for Predicting Metastatic Risk in Pheochromocytoma and Paraganglioma. Am. J. Surg. Pathol..

[6] Tatemoto K., Hosoya M., Habata Y., Fujii R., Kakegawa T., Zou M.X., Kawamata Y., Fukusumi S., Hinuma S., Kitada C. (1998). Isolation and characterization of a novel endogenous peptide ligand for the human APJ receptor. Biochem. Biophys. Res. Commun..

[7] Liu L., Yi X., Lu C., Wang Y., Xiao Q., Zhang L., Pang Y., Guan X. (2021). Study Progression of Apelin/APJ Signaling and Apela in Different Types of Cancer. Front. Oncol..

[8] Agarwala Y., Sharma R. (2025). The role of apelin in cancer. Front. Oncol..

[9] Naldi L., Peri A., Fibbi B. (2025). Apelin/APJ: Another Player in the Cancer Biology Network. Int. J. Mol. Sci..

[10] Sorli S.C., Le Gonidec S., Knibiehler B., Audigier Y. (2007). Apelin is a potent activator of tumour neoangiogenesis. Oncogene.

[11] Pérez-Gutiérrez L., Ferrara N. (2023). Biology and therapeutic targeting of vascular endothelial growth factor A. Nat. Rev. Mol. Cell Biol..

[12] Shaw P., Dwivedi S.K.D., Bhattacharya R., Mukherjee P., Rao G. (2024). VEGF signaling: Role in angiogenesis and beyond. Biochim. Biophys. Acta Rev. Cancer.

[13] Mabeta P., Steenkamp V. (2022). The VEGF/VEGFR Axis Revisited: Implications for Cancer Therapy. Int. J. Mol. Sci..

[14] Raica M., Mogoantă L., Cîmpean A.M., Alexa A., Ioanovici S., Mărgăritescu C., Lazăr D., Izvernariu D. (2008). Immunohistochemical expression of vascular endothelial growth factor (VEGF) in intestinal type gastric carcinoma. Rom. J. Morphol. Embryol..

[15] Kang Y., Li H., Liu Y., Li Z. (2024). Regulation of VEGF-A expression and VEGF-A-targeted therapy in malignant tumors. J. Cancer Res. Clin. Oncol..

[16] Vimalraj S., Hariprabu K.N.G., Rahaman M., Govindasami P., Perumal K., Sekaran S., Ganapathy D. (2023). Vascular endothelial growth factor-C and its receptor-3 signaling in tumorigenesis. 3 Biotech.

[17] Ferrara N., Hillan K.J., Gerber H.P., Novotny W. (2004). Discovery and development of bevacizumab, an anti-VEGF antibody for treating cancer. Nat. Rev. Drug Discov..

[18] Gao X., Yamazaki Y., Pecori A., Tezuka Y., Ono Y., Omata K., Morimoto R., Nakamura Y., Satoh F., Sasano H. (2020). Histopathological Analysis of Tumor Microenvironment and Angiogenesis in Pheochromocytoma. Front. Endocrinol..

[19] Ferreira C.V., Siqueira D.R., Romitti M., Ceolin L., Brasil B.A., Meurer L., Capp C., Maia A.L. (2014). Role of VEGF-A and its receptors in sporadic and MEN2-associated pheochromocytoma. Int. J. Mol. Sci..

[20] Thompson L.D. (2002). Pheochromocytoma of the Adrenal gland Scaled Score (PASS) to separate benign from malignant neoplasms: A clinicopathologic and immunophenotypic study of 100 cases. Am. J. Surg. Pathol..

[21] Kimura N., Takayanagi R., Takizawa N., Itagaki E., Katabami T., Kakoi N., Rakugi H., Ikeda Y., Tanabe A., Nigawara T. (2014). Pathological grading for predicting metastasis in phaeochromocytoma and paraganglioma. Endocr. Relat. Cancer.

[22] Remmele W., Stegner H.E. (1987). Recommendation for uniform definition of an immunoreactive score (IRS) for immunohistochemical estrogen receptor detection (ER-ICA) in breast cancer tissue. Pathologe.

[23] Foersch S., Schmitt M., Litmeyer A.S., Tschurtschenthaler M., Gress T., Bartsch D.K., Pfarr N., Steiger K., Denkert C., Jesinghaus M. (2024). TROP2 in colorectal carcinoma: Associations with histopathology, molecular phenotype, and patient prognosis. J. Pathol. Clin. Res..

[24] Dzobo K., Senthebane D.A., Dandara C. (2023). The Tumor Microenvironment in Tumorigenesis and Therapy Resistance Revisited. Cancers.

[25] Magar A.G., Morya V.K., Kwak M.K., Oh J.U., Noh K.C. (2024). A Molecular Perspective on HIF-1α and Angiogenic Stimulator Networks and Their Role in Solid Tumors: An Update. Int. J. Mol. Sci..

[26] Srabovic N., Mujagic Z., Mujanovic-Mustedanagic J., Softic A., Muminovic Z., Rifatbegovic A., Begic L. (2013). Vascular endothelial growth factor receptor-1 expression in breast cancer and its correlation to vascular endothelial growth factor a. Int. J. Breast Cancer.

[27] Guillaume Z., Auvray M., Vano Y., Oudard S., Helley D., Mauge L. (2022). Renal Carcinoma and Angiogenesis: Therapeutic Target and Biomarkers of Response in Current Therapies. Cancers.

[28] Kiriakopoulos A., Giannakis P., Menenakos E. (2023). Pheochromocytoma: A changing perspective and current concepts. Ther. Adv. Endocrinol. Metab..

[29] Parikh P.P., Rubio G.A., Farra J.C., Lew J.I. (2017). Nationwide review of hormonally active adrenal tumors highlights high morbidity in pheochromocytoma. J. Surg. Res..

[30] Martinelli S., Amore F., Canu L., Maggi M., Rapizzi E. (2023). Tumour microenvironment in pheochromocytoma and paraganglioma. Front. Endocrinol..

[31] Liu Q., Djuricin G., Staren E.D., Gattuso P., Gould V.E., Shen J., Saclarides T., Rubin D.B., Prinz R.A. (1996). Tumor angiogenesis in pheochromocytomas and paragangliomas. Surgery.

[32] Ohji H., Sasagawa I., Iciyanagi O., Suzuki Y., Nakada T. (2001). Tumour angiogenesis and Ki-67 expression in phaeochromocytoma. BJU Int..

[33] Salmenkivi K., Heikkilä P., Liu J., Haglund C., Arola J. (2003). VEGF in 105 pheochromocytomas: Enhanced expression correlates with malignant outcome. Apmis.

[34] Zielke A., Middeke M., Hoffmann S., Colombo-Benkmann M., Barth P., Hassan I., Wunderlich A., Hofbauer L.C., Duh Q.Y. (2002). VEGF-mediated angiogenesis of human pheochromocytomas is associated to malignancy and inhibited by anti-VEGF antibodies in experimental tumors. Surgery.

[35] Abe I., Islam F., Lo C.Y., Liew V., Pillai S., Lam A.K. (2021). VEGF-A/VEGF-B/VEGF-C expressions in non-hereditary, non-metastatic phaeochromocytoma. Histol. Histopathol..

[36] Kocabaş M., Can M., Karaköse M., Esen H.H., Kulaksizoğlu M., Karakurt F. (2023). Expression of endocan and vascular endothelial growth factor and their correlation with histopathological prognostic parameters in pheochromocytoma. Endocrine.

[37] Günler T., Çordan İ. (2026). Increased HIF-2α and CD105 (endoglin) expression is associated with poor differentiation in pheochromocytomas and paragangliomas. Front. Endocrinol..

[38] Zhu Y., Zhang P., Huo X., Ling Y., Lv X., Lin S., Song H. (2024). Single-cell and spatial transcriptomics reveal apelin/APJ pathway’s role in microvessel formation and tumour progression in hepatocellular carcinoma. J. Cell Mol. Med..

[39] Wu L., Chen L., Li L. (2017). Apelin/APJ system: A novel promising therapy target for pathological angiogenesis. Clin. Chim. Acta.

[40] Kidoya H., Takakura N. (2012). Biology of the apelin-APJ axis in vascular formation. J. Biochem..

[41] Chen J., Li Z., Zhao Q., Chen L. (2022). Roles of apelin/APJ system in cancer: Biomarker, predictor, and emerging therapeutic target. J. Cell Physiol..

[42] Masoumi J., Jafarzadeh A., Khorramdelazad H., Abbasloui M., Abdolalizadeh J., Jamali N. (2020). Role of Apelin/APJ axis in cancer development and progression. Adv. Med. Sci..

[43] Wang Y., Chen D., Pang Y., Xu X., Guan X., Liu L. (2022). Value of Immunohistochemical Expression of Apelin, Succinate Dehydrogenase B, Chromogranin B, Human Epidermal Growth Factor Receptor-2, Contactin 4, and Succinyl-CoA Synthetase Subunit Beta in Differentiating Metastatic From Non-Metastatic Pheochromocytoma and Paraganglioma. Front. Endocrinol..

[44] Amar L., Servais A., Gimenez-Roqueplo A.P., Zinzindohoue F., Chatellier G., Plouin P.F. (2005). Year of diagnosis, features at presentation, and risk of recurrence in patients with pheochromocytoma or secreting paraganglioma. J. Clin. Endocrinol. Metab..

[45] Shirali A.S., Clemente-Gutierrez U., Huang B.L., Lui M.S., Chiang Y.J., Jimenez C., Fisher S.B., Graham P.H., Lee J.E., Grubbs E.G. (2023). Pheochromocytoma recurrence in hereditary disease: Does a cortical-sparing technique increase recurrence rate?. Surgery.

[46] Jung S., Shin H.R., Shin S.J., Na H.Y., Hong S.W., Park S.Y., Jung C.K., Jung K.C., Oh Y.L., Won J.K. (2026). Multicenter evaluation of the PASS score as a negative predictive tool and the impact of inter-observer variability in pheochromocytoma and paraganglioma risk stratification. J. Pathol. Transl. Med..

[47] de Bresser C.J.M., de Krijger R.R. (2024). The Molecular Classification of Pheochromocytomas and Paragangliomas: Discovering the Genomic and Immune Landscape of Metastatic Disease. Endocr. Pathol..

[48] Respekta N., Pich K., Dawid M., Mlyczyńska E., Kurowska P., Rak A. (2022). The Apelinergic System: Apelin, ELABELA, and APJ Action on Cell Apoptosis: Anti-Apoptotic or Pro-Apoptotic Effect?. Cells.

